# Supplementary material for: A high-resolution mRNA expression time course of embryonic development in zebrafish
Source: eLife. 2017 Nov 16;6:e30860. doi: 10.7554/eLife.30860 (PMC5690287; doi:10.7554/eLife.30860)
Supplement: Supplementary file 6. [file elife-30860-supp6.zip › biolayout-clusters-files/Cluster246.html]

Cluster246


# Cluster246: Detail

### Go to ZFA detail

## GO

| GO ID | Description | Domain | Annotated | Expected | Observed | Adjusted p-value | Genes | Ensembl IDs |
| --- | --- | --- | --- | --- | --- | --- | --- | --- |
| GO:0005634 | nucleus | cellular\_component | 1915 | 1.4 | 6 | 0.036 | psmd11b vcp psmd12 tada3l eef1e1 psma3 | ENSDARG00000005134 ENSDARG00000020008 ENSDARG00000052377 ENSDARG00000058220 ENSDARG00000071774 ENSDARG00000086618 |
